# Design and Manufacturing of Si-Based Non-Oxide Cellular Ceramic Structures through Indirect 3D Printing

**DOI:** 10.3390/ma15020471

**Published:** 2022-01-08

**Authors:** Ghenwa El Chawich, Joelle El Hayek, Vincent Rouessac, Didier Cot, Bertrand Rebière, Roland Habchi, Hélène Garay, Mikhael Bechelany, Mirvat Zakhour, Philippe Miele, Chrystelle Salameh

**Affiliations:** 1 Institut Européen des Membranes, IEM, UMR 5635, University Montpellier, CNRS, ENSCM, CEDEX 5, 34095 Montpellier, France; ghenwa-f-chawich-22@outlook.com (G.E.C.); joelle.el-hayek@etu.umontpellier.fr (J.E.H.); vincent.rouessac@umontpellier.fr (V.R.); didier.cot@umontpellier.fr (D.C.); mikhael.bechelany@umontpellier.fr (M.B.); philippe.miele@umontpellier.fr (P.M.); 2 Laboratoire de Chimie Physique des Matériaux/Plateforme de Recherche en Nanomatériaux et Nanotechnologies (LCPM/PR2N), Lebanese University, Beirut 90656, Lebanon; rhabchi@ul.edu.lb (R.H.); mzakhour@ul.edu.lb (M.Z.); 3 Institut Charles Gerhardt Montpellier (ICGM), UMR 5253, University Montpellier, CNRS, ENSCM, CEDEX 5, 34095 Montpellier, France; bertrand.rebiere@umontpellier.fr; 4 Institut des Sciences Analytiques et de Physico-Chimie pour l’Evironnement et les Matériaux (IPREM), IMT Mines Alès, Université de Pau et des Pays de l’Adour, E2S UPPA, CNRS, 64053 Pau, France; helene.garay@mines-ales.fr; 5 Institut Universitaire de France, IUF, MENESR, 1 rue Descartes, CEDEX 5, 75231 Paris, France

**Keywords:** additive manufacturing, Fused Deposition Modeling, Polymer-Derived Ceramics, non-oxide ceramics, replica process, SiCN, SiC

## Abstract

Additive manufacturing of Polymer-Derived Ceramics (PDCs) is regarded as a disruptive fabrication process that includes several technologies such as light curing and ink writing. However, 3D printing based on material extrusion is still not fully explored. Here, an indirect 3D printing approach combining Fused Deposition Modeling (FDM) and replica process is demonstrated as a simple and low-cost approach to deliver complex near-net-shaped cellular Si-based non-oxide ceramic architectures while preserving the structure. 3D-Printed honeycomb polylactic acid (PLA) lattices were dip-coated with two preceramic polymers (polyvinylsilazane and allylhydridopolycarbosilane) and then converted by pyrolysis respectively into SiCN and SiC ceramics. All the steps of the process (printing resolution and surface finishing, cross-linking, dip-coating, drying and pyrolysis) were optimized and controlled. Despite some internal and surface defects observed by topography, 3D-printed materials exhibited a retention of the highly porous honeycomb shape after pyrolysis. Weight loss, volume shrinkage, roughness and microstructural evolution with high annealing temperatures are discussed. Our results show that the sacrificial mold-assisted 3D printing is a suitable rapid approach for producing customizable lightweight highly stable Si-based 3D non-oxide ceramics.

## 1. Introduction

Ceramic materials, due to their superior properties such as high hardness, high melting point, low density and environmental tolerance, play an important role in the development of cutting-edge technologies. In particular, ceramic components with specific shapes and functions are required especially for electronics, aerospace, biomedical engineering and various other technological domains [1,2,3,4,5]. On-demand customizability of ceramics is therefore needed [6,7]. Due to the poor machinability of ceramics, complex structures are difficult to obtain using conventional techniques such as powder pressing, tape casting, freeze casting, molding, etc. [8]. Three-dimensional printing, also known as additive manufacturing (AM) or rapid prototyping, is an advanced manufacturing concept that can be advantageously used for producing near-net-shaped complex ceramic components [9]. Powders and slurries are usually the raw materials used for ceramics 3D printing. However, they exhibit many shortcomings such as inhomogeneous dispersion of ceramic particles in solution, sedimentation, low dimensional stability and inhomogeneity of the 3D printed material. As an alternative to conventional raw materials, preceramic polymers have been recently used in 3D printing of ceramics [10,11]. Preceramic polymers are a class of precursors that transform into ceramics upon pyrolysis at high temperature under inert or reactive atmosphere [12]. In the last years, Polymer-Derived Ceramics (PDCs)—including SiCN and SiC—have been attracting a lot of attention as high strength materials for several applications under harsh environments due to their unique multiphase structure, tailorable chemical composition, high thermal and chemical stability, excellent corrosion and oxidation resistance and designable shape [13,14,15,16,17,18]. Material extrusion, one of the most affordable 3D printing technologies, allows objects to be created by extruding the materials through a heated nozzle to build up a layer that instantly hardens so that the next layer adheres on top in a repeated process, ultimately leading to the formation of a 3D object. This process, also called Direct Ink Writing (DIW), allows multi-material printing of various colors at low-cost [19]. Fused Deposition Modeling (FDM) is the first extrusion process for thermoplastic polymers developed in the 1990s by Stratasys [20]. This technology is also referred as Fused Filament Fabrication (FFF) and Plastic Jet Printing (PJP). Although FDM is the easiest, most affordable and straightforward AM technology, it was rarely used for printing preceramic polymers. This can be related to the relatively high ceramic yield of Si-based preceramic polymers and their high glass transition temperature [12] which make them unsuitable for FDM [21]. Recently, several works were conducted on direct FDM of preceramic polymers, either by modifying the preceramic polymer with organic elastomeric binders [22] or by mixing it with thermoplastic polymers [23]. An alternative approach consists in using a polymer structure as a preform or a mold to fill with the preceramic polymer which was demonstrated on SiOC by Kulkarni et al. [24]. However, the work dealt with oxide ceramics and the coating occurred in presence of a platinum-based catalyst thus limiting the scalability of the process.

In our research, we are interested in non-oxide silicon-based Polymer-Derived ceramics (PDCs) known for having great thermochemical stability, high mechanical properties, oxidation resistance, chemical resistance, high melting point, wear resistance and high hardness compared to their oxide homologues [13,16,25,26,27,28]. They are also explored as a versatile class of materials for various applications in catalysis, energy, machinery and biomedical engineering [8]. Therefore, structural design and fabrication of 3D non-oxide PDCs are highly desired. So far, 3D printing of non-oxide ceramics has been mostly performed with methods based on photopolymerization such as Stereolithography (SLA) and Digital Light Processing (DLP) [11]. In order to be UV-curable, liquid polysilazane and polycarbosilane were chemically modified with photosensitive functionalities [29,30] or simply mixed with photosensitive resins [31]. For example, photopolymerization of hyperbranched polycarbosilane [32] and allylmethylhydridopolycarbosilane [33] was realized using SLA to form SiC ceramics, after a proper heat treatment. 3D SiCN ceramics were printed through photopolymerisation of polysilazane [1,34,35]. Similarly, multicomponent Si-based carbonitrides, i.e., SiBCN were obtained through DLP 3D printing [31,36]. Nevertheless, further research efforts are needed to enlarge the additive manufacturing possibilities and propose a reliable process applicable for a large panel of PDCs and for ceramics in general.

As AM industries and AM end users explore technical and high-performance materials to process additively, a few companies have begun exploring silicon carbide 3D printing by using powder bed, extrusion and photopolymerization-type processes and are starting to get interested in extrusion-based 3D printing due to its low-cost and accessibility. For example, one of the first firms to explore silicon carbide 3D printing was HRL Laboratories by developing preceramic resins that can be cured with ultraviolet light in commercially available stereolithography 3D printers or through a patterned mask. Our proposed approach and derived 3D non-oxide ceramics can thus be attracting from an industrial point of view as it proposes low-cost and accessible extrusion-based technology that can be adapted for a large panel of ceramic compositions. One of the most common uses in industrial manufacturing is automotive parts, such as brake discs as well as electronics, heating elements and catalytic converters. Porous ceramics are also interesting for applications in defense and biomedical fields. In the defense sector, the cellular materials can be used as structural support for the core of sandwich composites designed to resist high amounts of dynamic loads. For biomedical applications, the porosity in scaffolds and lattice structures can be engineered to fulfil the mechanical properties and biodegradability required to replace the damaged bones.

Usually, the presence of pores within the ceramics can critically affect the mechanical and fatigue strength, corrosion resistance, stiffness and fracture toughness properties of the ceramics. Our approach can be employed in the manufacturing of ceramic components with various porous structure through topology optimization process of scaffolds and lattices to improve their toughness. The undesirable effect of pores can be eliminated by (1) using preceramic polymers with high ceramic yield and crosslinking agents, (2) optimizing the processing parameters and (3) implementing suitable post-processing thermal treatment. Dense ceramics are however difficult to obtain through this method due to the limited infiltration of the preceramic solutions into a polymer dense preform. For printing dense 3D ceramics UV polymerization-based technologies are more appropriate.

In this work, we chose a low-cost and simple approach based on combining FDM 3D printing with the replica technique—i.e., through the use of a sacrificial preform—and applied it on two non-oxide Si-based ceramics with complex cellular shapes. First, cellular honeycomb structures of polylactic acid (PLA) were printed using FDM. Second, dip-coating of PLA molds was performed with catalyst-free PolyVinylsilaZane (PVZ) and AllylHydridoPolyCarboSilane (AHPCS) precursors of SiCN and SiC, respectively. A subsequent pyrolysis under inert and controlled atmosphere was performed on the composites to non-invasively decompose the PLA preform while ensuring the polymer-to-ceramic transformation. Geometric stability and shape retention capacity were demonstrated by microscopy tools. Thermostructural tenacity of the 3D ceramics was probed by X-ray diffraction and Raman spectroscopy.

Production of 3D SiC(N) ceramics with controlled geometries by this facile two-step method brings new insights in the additive manufacturing of ceramics. Using commercial, low-cost and available thermoplastic polymers as the preform’s material and reducing the quantity of preceramic polymers for coating without the use of catalysts reduce the overall manufacturing process.

## 2. Materials and Methods

### 2.1. Reagents and Materials

PolyVinylSilazane (abbreviated PVZ, also called durazane, Merck, Darmstadt, Germany), AllylHydridoPolyCarboSilane (abbreviated AHPCS, also called SMP-10, Starfire, East Glenville, NY, USA), Toluene (anhydrous 99.8%, Merck, Darmstadt, Germany) and DiCumyl Peroxide (abbreviated DCP, C_18_H_22_O_2_, Merck, Darmstadt, Germany) were used as received without further purification. Potassium Bromide KBr (99%, Merck, Darmstadt, Germany) was dried overnight at 120 °C prior to the pellet preparation for Fourier Transform Infrared (FT-IR) spectroscopy. Commercial PolyLactic Acid (PLA) 1.75 mm diameter transparent filaments were purchased from Prusa Research, Prague, Czech Republic. The gases used for pyrolysis are argon (99.999%) and nitrogen (99.995%) and were purchased from Linde, France.

### 2.2. Preparation of Preceramic Polymer Mixtures

All chemicals, mixing and coating steps were handled in an argon filled glovebox (Jacomex, O_2_ and H_2_O < 0.5 ppm) and using Schlenk techniques on a vacuum/argon line (with argon purified through a Siccapent phosphorus pentoxide column and BTS catalyst). 3 wt% of dicumyl peroxide were dissolved in PVZ and AHPCS solutions to obtain DCP-PVZ and DCP-AHPCS, respectively.

### 2.3. 3D Printing of Honeycomb Structures

3D printing of PLA filaments with 1.75 mm diameter was conducted on a FDM Prusa i3 MK2S printer (Prusa Research, Prague, Czech Republic). PLA molds were designed as 10 mm × 6 mm cylindrical honeycomb cellular structures and 15 mm × 15 mm × 6 mm square cellular structures and were printed layer by layer with 25% infill density, a first layer printing speed of 20 mm/s, filling speed of 60 mm/s and a perimeter of 1 using the Prusa slicer software (version 2.4) from Prusa Research, Prague, Czech Republic. The printing temperature was fixed at 240 °C. Note that several parameters including the printing orientation, printing temperature, speed, infill style and percentage can improve the quality and resolution of the printed objects as reported by Zhou et al. [37].

### 2.4. Dip-Coating of PLA Molds with Preceramic Polymer Solutions

After 3D printing, PLA molds were repeatedly dipped and soaked for 20 s in DCP-PVZ and DCP-AHPCS solutions then they were left to dry for 30 min leading, respectively, to composites labeled PLA/DCP-PVZ and PLA/DCP-AHPCS. Each cycle was repeated 6 times using a dip-coater (Ossila, Sheffield, UK). This sequence has proven to be useful for preserving the 3D structure of the initial mold after thermal treatment. The immersion and withdrawal speeds of dip-coating were set to 20 mm/s and 10 mm/s respectively.

### 2.5. Polymer-to-Ceramic Conversion

Impregnated PLA molds were pyrolyzed in a tubular furnace (type ROS20/250/12 with a silica tube, Thermconcept, Bremen, Germany) at 1000 °C under inert atmosphere (nitrogen for SiCN ceramic and argon for SiC ceramic) according to the following heat treatment: (i) crosslinking of the preceramic polymers with DCP at 130 °C for 2 h (heating rate 2 °C∙min^−1^), (ii) decomposition of PLA sacrificial mold at 250 °C for 1 h then at 320 °C for 5 h (heating rate of 1 °C∙min^−1^) and (iii) polymer-to-ceramic conversion at 1000 °C for 2 h (heating and cooling rate of 2 °C∙min^−1^).

The 3D ceramics were subsequently annealed at high temperatures (1200–1700 °C) for 2 h under inert atmosphere (nitrogen for SiCN and argon for SiC) in a graphitic furnace (Gero, Model HTK8, Carbolite Gero, Neuhausen, Germany) in order to investigate the crystallization of the ceramics. During all the thermal treatments, the materials were loaded in the furnace, after which the furnace chamber was evacuated for 1 h then filled with gas with a 200 mL∙min^−1^ flow rate during the treatment.

### 2.6. Characterization

Thermogravimetric analysis of all polymers was conducted on a TGA-SDT Q600 thermal analysis device (TA Instruments, New Castle, DE, USA) at atmospheric pressure from RT up to 1000 °C with a heating rate of 5 °C∙min^−1^ under an inert atmosphere. The chemical structure of the preceramic polymers was determined by Fourier Transform Infrared (FT-IR) spectroscopy with a Nicolet iS50 Thermo scientific spectrometer, ThermoFisher Scientific, Waltham, MA, USA, using KBr pellets prepared in the glove box (2 wt% of polymer mixed with previously dried KBr powder followed by compaction into a dense pellet). The morphology of pyrolyzed 3D lattices was observed by the super depth field three-dimensional optical microscope (VHX-7000, KEYENCE, Osaka, Japan) and Scanning Electron Microscopy (Hitachi S4800, Hitachi High-Tech Corporation, Tokyo, Japan, operating with an acceleration voltage of 2 kV). The chemical composition of SiCN and SiC 3D ceramics was analyzed using Energy Dispersive X-ray spectroscopy (Detector: Oxford Instruments X-Max N SDD, Abingdon, UK; Microscopy: Zeiss EVO HD15, Jena, Germany). The evolution of the microstructure of SiCN and SiC 3D ceramics between 1000 and 1700 °C was monitored by ex situ X-ray diffraction and Raman spectroscopy. X-ray diffraction was performed on a PANAlytical X’pert-PRO diffraction system (Malvern Panalytical, Almelo, Netherlands) operating at 20 mA and 40 kV from 10 to 90° with a step size of 0.0167, using a Kα1 of copper as source λ = 0.154 nm. Raman spectroscopy was carried out through Renishaw inVia Raman microscope (Gloucestershire, UK) equipped with an objective (Leica, ×50) using a green laser light of 532 nm. The laser power was kept below 1.5 mW to protect the sample from laser damage. Further, to ensure the quality of the Raman spectroscopic signal, we averaged 3 scans per spectrum and smoothed and baseline corrected all spectra using the Renishaw WiRE (version 5.2, Wotton-under-Edge, United Kingdom) software for all the measurements. The topography of the ceramic surfaces was measured on 15 mm × 15 mm × 6 mm square cellular structures by a confocal chromatic roughness apparatus (STIL SA, Aix en Provence, France) composed of a controller CCS Prima, a CHR1000 sensor and a displacement table Micromesure controlled with SurfaceMap acquisition software. Two different locations of 2 mm × 2 mm for each mold, with a 5 µm lateral step were measured. Postprocessing of the data was done with MontainsMap v7 software (DigitalSurf, Besançon, France). The bulk density of the 3D honeycombs was calculated from the weight and volume of the samples by the Archimedes method and the porosity deduced.

## 3. Results

### 3.1. Characterization of Preceramic Polymers

**Figure 1:**
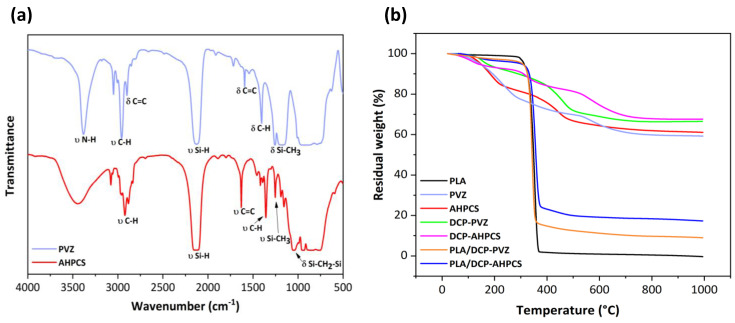
(**a**) FT-IR spectra of AHPCS and PVZ. (**b**) TGA plots recorded between room temperature and 1000 °C under inert atmosphere on PVZ and AHPCS, DCP-PVZ and DCP-AHPCS, PLA molds coated with preceramic polymers i.e., PLA/DCP-PVZ and PLA/DCP-AHPCS.

The chemical structure of PVZ and AHPCS preceramic polymers was characterized by FT-IR spectroscopy as shown in Figure 1a. As expected, PVZ displays all the conventional absorption bands of the different chemical functions such as C-H (1410 cm^−1^, 2963 cm^−1^), Si-H (2130 cm^−1^), Si–CH=CH_2_ (1588 cm^−1^, 2901 cm^−1^), Si-CH_3_ (1258 cm^−1^) and particularly N–H bonds at 3380 cm^−1^ coupled to the vibration of Si–N at 1170 cm^−1^ [38,39,40]. In the case of AHPCS, according to the literature, peaks in the spectrum are assigned to: C–H (1355 cm^−1^, 2932 cm^−1^), Si–H (2128 cm^−1^), Si–CH_2_–Si (1041 cm^−1^) and C=C (1622 cm^−1^) [14,41].

Polymer-to-ceramic conversion was investigated by means of TGA under inert atmosphere for both systems. Figure 1b reports TGA curves of all the components involved in the process: (i) PLA filament before printing the preform; (ii) PVZ and AHPCS without crosslinking; (iii) PVZ and AHPCS with 3 wt% DCP, namely, DCP-PVZ and DCP-AHPCS; and (iv) PLA preform coated with the crosslinkable preceramic polymers, namely, PLA/DCP-PVZ and PLA/DCP-AHPCS. PLA starts decomposing around 250 °C and shows a total decomposition at 320 °C, which is why we fixed a dwelling time relatively high at these particular temperatures during pyrolysis. PVZ shows three weight losses: The first weight loss around 250 °C (23%) corresponds to the volatilization of low molecular weight organosilicon species [39] and to crosslinking reactions of the preceramic polymer through transamination and bond redistribution as well as crosslinking via polymerization of vinyl groups and hydrosilylation reactions [42]. Dihydrogen is also considered as a gaseous by-product, probably due to dehydrocoupling reaction between ≡SiH/≡SiH and ≡SiH/=NH groups [39,43,44]. Thermal crosslinking of preceramic polymers takes place before the onset decomposition of PLA. The second mass loss occurring between 250 °C and 450 °C (7%) corresponds to transamination reactions by the NH groups in PVZ removing the ammonia. The third weight loss (11%) is due to the conversion of PVZ into SiCN material [39]. Similarly, AHPCS exhibits three weight losses. The first weight loss at ~250 °C (18%) is due to the volatilization of oligomers. The second weight loss between 250 °C and 450 °C (16%) corresponds to radical reactions releasing gaseous species such as methane, ethylene and propylene and the third weight loss (4%) is attributed to the transformation of AHPCS into SiC ceramic [14,45]. As expected, the ceramic yields of PVZ and AHPCS are relatively high: 59% and 62% respectively, which is a key factor for preserving the honeycomb structure after pyrolysis. Adding 3% of DCP to promote the crosslinking of preceramic polymers increases the ceramic yield by 10% as shown from TGA curves of DCP-PVZ and DCP-AHPCS. TGA curves of PLA honeycombs coated with preceramic polymers show a weight loss of 86% and 82% for PLA/DCP-PVZ and PLA/DCP-AHCPS respectively with a main decomposition step at ~320 °C. Such high values are attributed to the degradation of PLA. The use of dicumyl peroxide tends to chemically crosslink the preceramic polymer and harden the 3D honeycomb structure thus avoiding the loss of its structural integrity during thermal decomposition of the PLA.

After 3D printing, coating and pyrolysis, the weight losses reach 87% and 83% for SiCN and SiC 3D ceramics respectively, which is in agreement with the mass losses obtained by the TGA of PLA/DCP-PVZ and PLA/DCP-AHPCS. The weight loss is associated to the ceramization of preceramic polymer coatings on the 3D honeycomb structures and to the decomposition of PLA, proving the rational choice of the number of cycles during the dip-coating process [24].

As a control experiment, dip-coating of PLA honeycomb preform with PVZ and AHPCS was performed without using DCP and the structure was found to collapse after pyrolysis. This highlights the benefit of using DCP as crosslinking agent for a good retention of the 3D shape of the ceramics.

### 3.2. Characterization of SiCN and SiC Ceramics

#### 3.2.1. Morphological Properties

**Figure 2:**
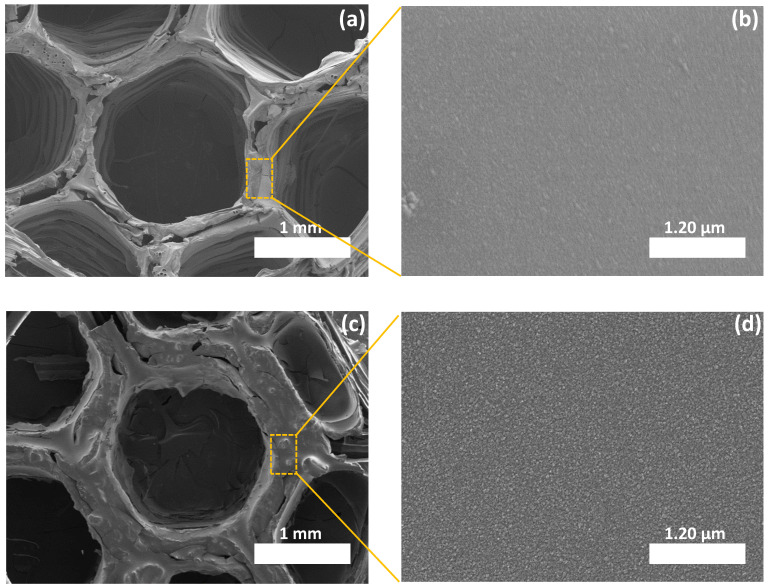
(**a**) SEM image of a SiCN cell. (**b**) Magnified image of a SiCN single wall. (**c**) SEM image of a SiC cell. (**d**) Magnified image of a SiC single wall.

After 3D printing the molds and the subsequent dip-coating and curing, derived 3D ceramic honeycombs were characterized by SEM and 3D optical microscopy to investigate their morphology and the shrinkage occurring during pyrolysis. As seen from SEM images (Figure 2a,c), the ceramics inherited the 3D cellular shape of PLA. Few cracks in the surface and between the walls of the honeycombs can be detected, especially in the case of SiCN. Such defects are the result of the decomposition of PLA and the fast escape of volatile species during ceramization. No obvious deformation of the structure was observed even though no filler or binder was used. This is due to the efficient coating of PLA by the PDC precursors and the appropriate thermolysis treatment.

The magnification on a wall of a honeycomb shows that the material has a dense texture with no sign of porosity (Figure 2b,d). The volume shrinkage measured after pyrolysis at 1000 °C are presented in Table 1. The shrinkage observed on our SiC(N) honeycombs derived from FDM-printed mold is similar to that resulting from SiCN derived from SLA-printed mold [46]. This result confirms the efficient ceramization of the coated lattices. The volume shrinkage (honeycomb cylinder diameter, pore diameter and honeycomb cylinder thickness) is higher in the case of 3D SiCN according to the slightly lower ceramic yield of PVZ compared to AHPCS.

**Figure 3:**
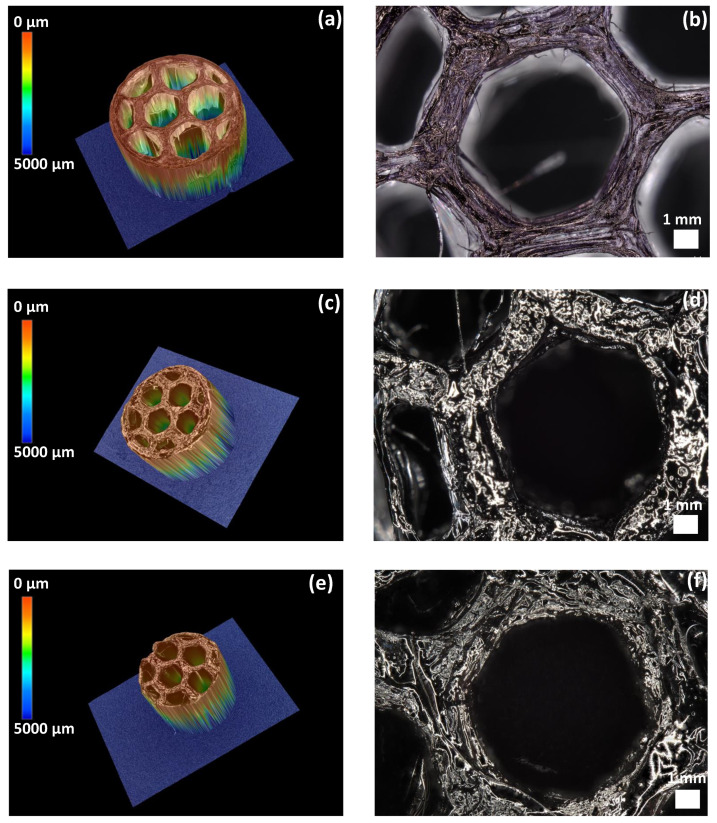
3D Optical microscopy images of (**a**,**b**) PLA 3D cellular structure. (**c**,**d**) 3D SiCN. (**e**,**f**) 3D SiC.

To further investigate the cellular structure of the materials, we carried out 3D optical microscopy on the PLA mold (Figure 3a,b), SiCN (Figure 3c,d) and SiC-derived ceramics (Figure 3e,f). Comparing SiCN and SiC ceramic structures with that of PLA, we observe a near-perfect preservation of the honeycomb structure. However, ~40% and ~20% decreases in the pore diameter and in the wall thickness, respectively, were identified after thermal treatment during which PLA decomposition and polymer-to-ceramic conversion simultaneously occurred. Overall, based on Figure 3, decreases in the whole mold diameter, mold thickness and pore diameter were observed before and after thermal treatment when comparing the dimensions of the 3D printed PLA to SiCN and SiC ceramics. Furthermore, a higher volume shrinkage can be noticed in the case of SiCN ceramic when compared to SiC with presence of cracks, which is attributed to the lower ceramic yield of PVZ.

**Figure 4:**
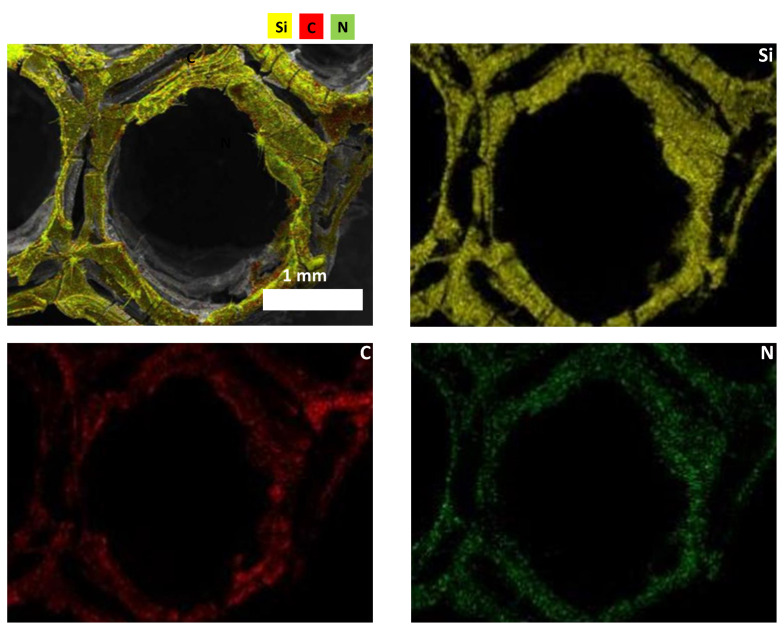
Elemental mapping of 3D SiCN.

**Figure 5:**
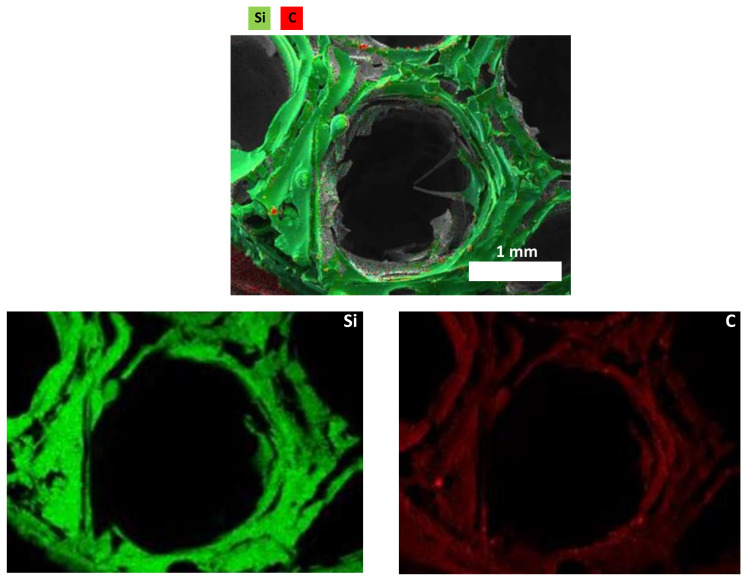
Elemental mapping of 3D SiC.

Elemental composition of the 3D printed ceramics was investigated by energy-dispersive X-ray diffraction (Table 2). These results indicate the effectiveness of the dip-coating process and the full decomposition of PLA while converting the preceramic polymers into ceramics. However, despite all the precautions taken (use of Schlenk lines, glove box and furnaces under inert atmospheres), a small quantity of oxygen can still be detected in both ceramics (3 wt%). This can be due to contamination while introducing the samples in the furnace for pyrolysis or to oxygen adsorption during EDX measurements since the ceramics were stored out of the glove box. Elemental mapping was performed on a single cell to show the homogeneous distribution of the elements on the material surface. The homogeneous distribution of the colors suggests that the elements of the ceramics, i.e., Si in yellow, C in red, N in green are uniformly dispersed as observed from the individual mapping of each element constituting the ceramic (Figure 4 for 3D SiCN and Figure 5 for 3D SiC). Oxygen mapping was not recorded due to its low presence.

#### 3.2.2. Microstructural Evolution

Amorphous 3D SiCN and SiC ceramic honeycombs were isothermally annealed at different temperatures (between 1000 and 1700 °C under inert atmosphere with 2 h hold-time). Qualitative and semi-quantitative studies of the crystallization behavior and microstructural evolution of the 3D materials were carried out by X-ray diffraction (XRD) and Raman spectroscopy.

**Figure 6:**
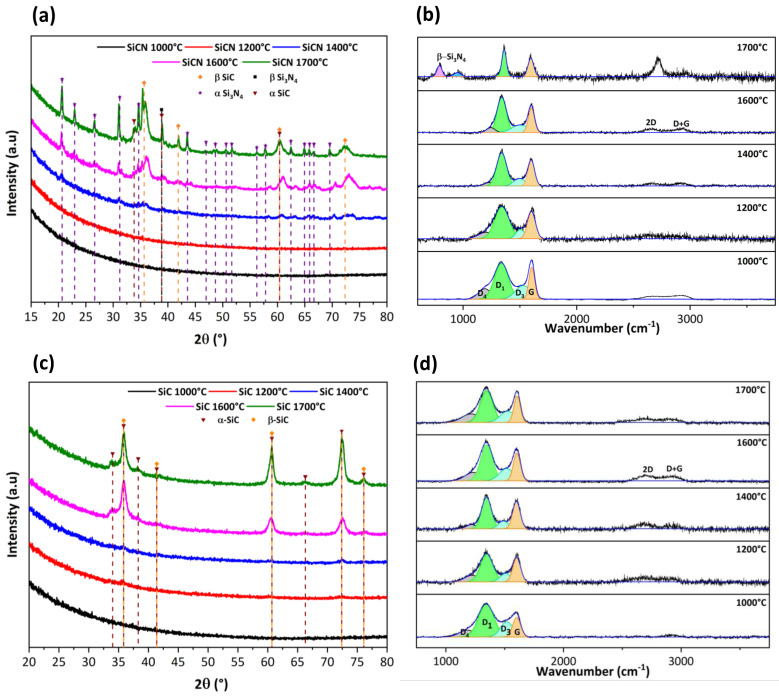
(**a**) XRD patterns of 3D SiCN. (**b**) Raman spectra of 3D SiCN. (**c**) XRD patterns of 3D SiC. (**d**) Raman spectrum of 3D SiC with temperatures ranging between 1000 and 1700 °C under inert atmosphere.

As expected, X-ray diffractograms show an amorphous phase for both ceramics at 1000 °C with no distinct crystalline peaks (Figure 6a,c). The diffractogram of 3D SiCN shows a slight crystallization that starts to appear at 1400 °C corresponding to SiC that is the first phase to nucleate (α-SiC hexagonal 2ϴ = 33.81° (1 0 0); 38.86° (1 0 2); 60.38° (1 1 0) and β-SiC cubic at 2ϴ = 35.90° (1 1 1); 41.35° (2 0 0); 60.75° (2 2 0), 72.45° (3 1 1) and 76.03° (2 2 2) phases). At and above 1600 °C, peaks of hexagonal α-Si_3_N_4_ appear at 2ϴ = 20.60° (1 0 1); 22.96° (1 1 0); 26.62° (2 0 0); 31.19° (2 0 1); 34.65° (1 0 2); 43.50° (3 0 1); 46.96 (2 2 0); 48.76° (3 1 0); 50.62° (1 0 3); 51.66° (−3 −1 1); 56.21° (2 0 3); 57.82° (−2 −2 2); 62.45° (JCPDS 01-071-6479) as well as β-Si_3_N_4_ hexagonal at 2ϴ = 38.86° (1 1 1) (JCPDS 01-082-0704). Crystallization indeed accelerates with increasing temperature.

Similarly, 3D SiC remains amorphous up to 1400 °C. The diffractogram of 3D SiC at 1600 °C shows the presence of very diffuse crystalline peaks corresponding to the planes diffraction of the cubic β-SiC phase at 2ϴ = 35.90° (1 1 1); 41.35° (2 0 0); 60.75° (2 2 0), 72.45° (3 1 1) and 76.03° (2 2 2) (JCPDS 00-002-1050) as well as the hexagonal α-SiC phase at 2ϴ = 34.04° (1 0 1); 35.90° (0 0 4); 38.23° (1 0 2); 41.35° (1 0 3); 60.75° (1 1 0); 66.26° (1 0 6); 72.45° (1 1 4); and 76.03° (2 0 2) (JCPDS 00-022-1317). These data are consistent with the results obtained in the literature for the conversion of polysilazane into SiCN [38] and polycarbosilane into SiC [47,48,49] which practically means that the 3D printing replica process did not alter the intrinsic microstructure of the ceramics.

In order to study the structural composition of carbon in SiCN and SiC 3D lattices, Raman spectroscopy was performed (Figure 6b,d). Four bands—D_1_, D_3_, D_4_ and G—were detected after deconvolution of the peaks. The G band corresponds to defect-free sp^2^ carbon arrays with a symmetric E_2_g vibrational mode. The D_1_ band, with a symmetric vibration mode A_1_g, is associated with small crystallites or grains sizes or defects in the graphitic domains. The D_3_ band is attributed to amorphous carbon while the presence of the D_4_ band has been tentatively related to the existence of polyene-like structures or ionic impurities. The intensity ratio of D and G peaks (Id/Ig) is proportional to the in-plane correlation length. This ratio increases with decreasing size of the perfect graphene units, as the disorder increases and the D mode becomes more active [50,51,52]. Table 3 represents the position of the G band, the full width at half maximum FWHM (cm^−1^) of the G band as well as the intensity ratio of the D_1_ and G bands denoted Id /Ig of the 3D ceramics at different temperatures ranging from 1000 °C to 1700 °C.

For 3D SiCN ceramic, the position of the G band as well as the FWHM decrease, respectively, from 1604 cm^−1^ to 1596 cm^−1^ and from 90 cm^−1^ to 60 cm^−1^, indicating that the free carbon phase changes from amorphous carbon to crystalline graphite [38]. Id/Ig is used to calculate the nanocluster size of the free carbon; the increase of this ratio from 1.12 to 1.48—with temperature ranging from 1000 °C to 1700 °C—indicates the development of nanopolycrystalline graphite as already reported by Chen et al. [53]. The intensity of the 2D peaks increases with temperature indicating a progressive and continuous increase of the crystallinity accompanied with a total disappearance of the D_4_ band and almost total disappearance of the D_3_ band suggesting a decrease of defects in the structure by pyrolysis at high temperature. At 1700 °C, the appearance of the two peaks at approximately 800 cm^−1^ and 945 cm^−1^ corresponds to the β-Si_3_N_4_ phase [54].

For 3D SiC ceramic, the change in the position of the G-band is not observed when heating between 1000 °C to 1700 °C. On the other hand, the FWHM decreases from 80 cm^−1^ to 64 cm^−1^ indicating that within the free carbon phases, an ordering process has occurred; the peaks become narrower contributing to an increase in crystallinity with temperature. The Id/Ig ratio is related to the in-plane correlation length for crystalline and amorphous carbon. The intensity ratio first increased by going from 1000 °C to 1600 °C and then decreased at 1700 °C. Therefore, a temperature of 1600 °C is a critical value where the microstructure changes from amorphous carbon to graphite [48]. In addition, the intensity of the 2D peaks increases with temperature indicating a good crystallinity starting from 1600 °C. These results are consistent with XRD where the α-SiC and β-SiC phases are more intense.

#### 3.2.3. Evolution of the Porosity with the Temperature

**Figure 7:**
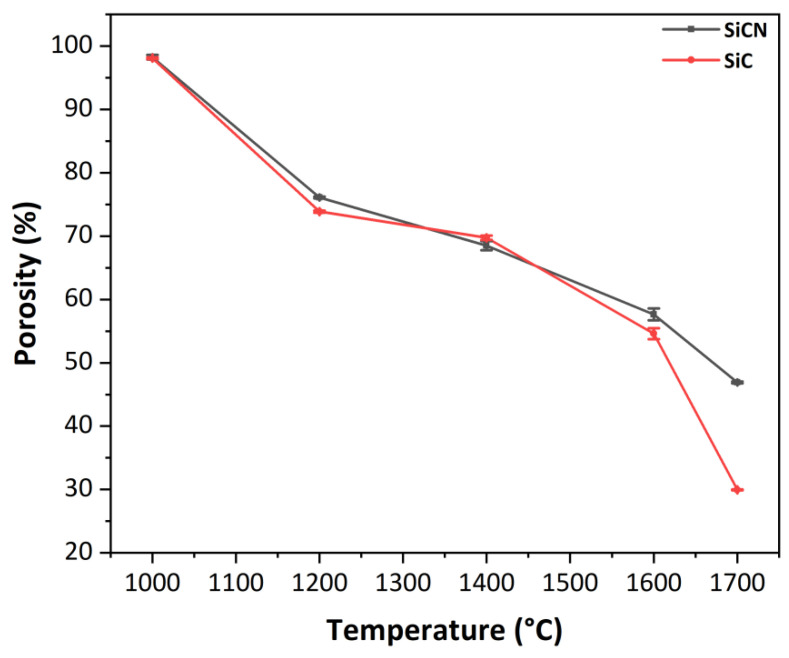
Evolution of the porosity of 3D SiCN and SiC ceramics with temperatures ranging between 1000 and 1700 °C under inert atmosphere.

The porosity of 3D ceramics obtained after heat treatment under inert atmosphere at temperatures ranging from 1000 °C to 1700 °C was calculated using the Archimedes principle in ethanol. Each value is an average of three porosity values calculated for each material at a fixed temperature T(°C). As the temperature increases from 1000 °C to 1700 °C, the densification increases and the porosity decreases by 51% and 68% for SiCN and SiC 3D ceramics respectively (Figure 7). This result is similar to that obtained by Chae et al. [55] where the porosity of SiC ceramic decreases when temperature rises from 1750 °C to 1900 °C.

Overall, after pyrolysis at 1000 °C under inert atmosphere, the 3D ceramics are very porous according to Archimedes calculations (~98%) and are robust enough for handling. As the thermolysis temperature increases from (1000 to 1700 °C), the porosity collapses and the ceramics tend to densify. This can be attributed to the tendency for the different systems to reduce their surface energy.

#### 3.2.4. Roughness of SiCN and SiC 3D Ceramics

**Figure 8:**
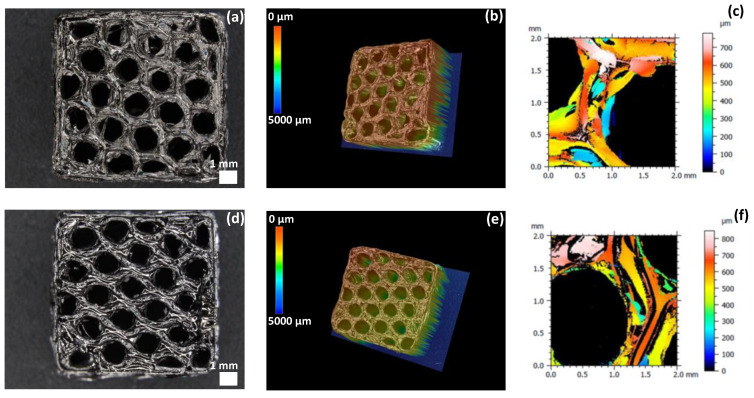
(**a**,**b**) 3D images of SiCN square ceramic lattice. (**c**) Roughness profile of a wall of 3D SiCN. (**d**,**e**) 3D images of SiC square ceramic lattice. (**f**) Roughness profile of a wall of 3D SiC.

Several shapes can be printed by coupling 3D-FDM printing technology with the replica technique. A 6 mm thickness, 15 mm × 15 mm square was printed by FDM using a 25% fill density. This shape is the most adapted for the topography analysis. Six cycles of dip-coating (20 s dwelling and 30 min drying) of printed PLA in DCP-PVZ and DCP-AHPCS lead after pyrolysis to 3D SiCN and SiC square lattices respectively as shown in Figure 8.

Surface roughness study was investigated on these 3D ceramics. Figure 8c,f represents the roughness profile of a wall of the two ceramics SiCN and SiC. Several roughness parameters were studied on a wall connecting two cells to determine the roughness of the 3D ceramic lattices. Sq is the quadratic standard deviation, Ssk represents the factor of asymmetry of the surface, Sku is the factor of flattening of the surface, Sa is the arithmetic standard deviation and Sz is the mean value of the distances between the five higher peaks and the five deeper valleys of the surface. The parameters are presented in Table 4 for SiCN and SiC ceramics and each value is averaged over four values. Both 3D ceramics exhibit deep pores, which translates into a negative value of Ssk with a narrow profile distribution (Sku > 3). 3D SiCN ceramic is rougher than 3D SiC as demonstrated by the larger values of Sq, Sa and Sz.

## 4. Conclusions

Advanced 3D non-oxide ceramics with complex architectures are currently regarded for various applications requiring stability under severe conditions. AM is the technology of choice to design near-net-shaped objects with a perfect control of the geometry and porosity at different scales. Preceramic polymers are suitable for 3D printing as they can be easily adapted to different AM technologies. In this work, we combined FDM 3D printing with a replica approach to fabricate complex geometrical Si-based ceramic objects. Characterization of preceramic polymers and the derived 3D ceramic objects were systematically presented. The influence of the preceramic polymers composition and structure on the 3D printing ability was highlighted. Polylactic acid (PLA) honeycomb patterns were first printed by FDM and dip-coated with chemically crosslinked polyvinylsilazane (PVZ) or allylhydrydopolycarbosilane (AHPCS) that are precursors of SiCN and SiC, respectively. After thermal treatment under inert atmosphere, SiCN and SiC 3D ceramics were obtained and characterized by SEM, EDX and 3D microscopy. The microstructure was evaluated using ex situ X-ray diffraction and Raman spectroscopy at increasing temperatures from 1000 °C to 1700 °C under inert atmosphere. The measurement of the roughness and the porosity was also discussed. SEM and 3D optical microscopy images show a near-perfect preservation of the initial shape of the PLA mold for SiCN and SiC 3D ceramics. The mappings show the presence of all the elements of the ceramics and their homogeneous distribution on the 3D honeycomb surface. The limited crystallization of these non-oxide 3D systems, as proven by XRD and Raman spectroscopy, results in a superior decomposition resistance even at high temperatures, whereas the porosity of both ceramics decreases with temperature due to an increase of the densification.

## Figures and Tables

**Table 1:** Dimensions of 3D PLA, SiCN and SiC ceramics as well as the shrinkage after pyrolysis.

	Honeycomb Cylinder Diameter		Pore Diameter		Honeycomb Cylinder Thickness	
	Measure (mm)	Shrinkage (%)	Measure (mm)	Shrinkage (%)	Measure (mm)	Shrinkage (%)
PLA	10.00	-	2.85	-	6.00	-
SiCN	6.35 ± 0.12	36.5	1.75 ± 0.01	38.6	4.65 ± 0.13	22.5
SiC	6.78 ± 0.16	32.2	1.78 ± 0.01	37.5	4.85 ± 0.12	19.2

**Table 2:** Elemental composition of the 3D ceramics (wt%).

Ceramic Composition	Si (wt%)	C (wt%)	N (wt%)	O (wt%)	Empirical Formula
SiCN	36.71 ± 1.91	37.54 ± 2.67	23.46 ± 1.48	2.27 ± 0.98	(Si_1_C_1.02_N_0.63_O_0.06_)
SiC	49.97 ± 1.81	46.78 ± 1.91	-	3.23 ± 0.51	(Si_1_C_0.93_O_0.06_)

**Table 3:** Position, FWHM and Id/Ig for SiCN and SiC 3D ceramics.

	SiCN Ceramic			SiCN Ceramic		
	Position (cm^−1^)	FWHM (cm^−1^)	Id/Ig	Position (cm^−1^)	FWHM (cm^−1^)	Id/Ig
1000 °C	1601	90	1.12	1604	80	1.67
1200 °C	1602	82	1.16	1602	78	1.19
1400 °C	1602	79	1.30	1600	75	1.16
1600 °C	1603	64	1.43	1601	71	1.28
1700 °C	1605	60	1.48	1596	64	1.21

**Table 4:** Surface roughness of ceramics.

Ceramics Roughness	SiCN	SiC
Sq (µm)	109.44 ± 15.54	107.46 ± 27.87
Ssk	−0.92 ± 0.26	−0.55 ± 0.61
Sku	4.57 ± 0.86	3.62 ± 1.53
Sz	589.40 ± 58.31	552.40 ± 189.75
Sa	80.50 ± 10.66	83.22 ± 27.28

## Data Availability

Not applicable.
